# Distinct Features of Gut Microbiota in High-Altitude Tibetan and Middle-Altitude Han Hypertensive Patients

**DOI:** 10.1155/2020/1957843

**Published:** 2020-11-21

**Authors:** Lu-lu Zhu, Zhi-jun Ma, Ming Ren, Yu-miao Wei, Yu-hua Liao, You-lu Shen, Shi-ming Fan, Lin Li, Qing-xia Wu, Zhong-shan Gao, Jing-fu Song, Yu-lan Ma

**Affiliations:** ^1^Department of Cardiology, Affiliated Hospital of Qinghai University, Xining 810001, China; ^2^Department of Surgical Oncology, Affiliated Hospital of Qinghai University, Xining 810001, China; ^3^Laboratory of Cardiovascular Immunology, Institute of Cardiology, Union Hospital, Tongji Medical College, Huazhong University of Science & Technology, Wuhan 430074, China; ^4^Department of Urology, Affiliated Hospital of Qinghai University, Xining 810001, China

## Abstract

Indigenous animals show unique gut microbiota (GM) in the Tibetan plateau. However, it is unknown whether the hypertensive indigenous people in plateau also have the distinct gut bacteria, different from those living in plains. We sequenced the V3-V4 region of the gut bacteria 16S ribosomal RNA (rRNA) gene of feces samples among hypertensive patients (HPs) and healthy individuals (HIs) from 3 distinct altitudes: Tibetans from high altitude (3600–4500 m, *n* = 38 and 34), Hans from middle altitude (2260 m, *n* = 49 and 35), and Hans from low altitude (13 m, *n* = 34 and 35) and then analyzed the GM composition among hypertensive and healthy subgroups using the bioinformatics analysis, respectively. The GM of high-altitude Tibetan and middle-altitude Han HPs presented greater *α*- and *β*-diversities, lower ratio of Firmicutes/Bacteroidetes (F/B), and higher abundance of beneficial Verrucomicrobia and Akkermansia than the low-altitudes HPs did. The GM of high-altitude Tibetan and middle-altitude HIs showed greater *α*-diversity and lower ratio of F/B than the low-altitudes HIs did. But, *β*-diversity and abundance of Verrucomicrobia and Akkermansia among different subgroups of HIs did not show any differences. Conclusively, the high-altitude Tibetan and middle-altitude Han HPs have a distinct feature of GM, which may be important in their adaptation to hypertension in the plateau environments.

## 1. Introduction

The pathogenesis of hypertension is complex and multifactorial. Based on the currently known neurohumoral pathogenic mechanisms and risk factors of hypertension, scientists have been continuing to explore additional promising risk factors and pathogenesis of hypertension from different aspects to well control blood pressure (BP). Recently, the GM has drawn considerable attractions. Relationships between GM and metabolic diseases, such as obesity, diabetes, and coronary heart disease, have been got numerous literature reports [1–3]. And, researches have shown the GM of the HPs differed significantly from that of HIs in terms of decreased microbial richness, evenness, and diversity, reduced beneficial strains, and increased harmful strains and higher ratio of F/B [4–6].

However, host's GM can be affected by various factors. Besides unchangeable internal genetic background [7], unique external geography environment characteristics, including regional altitude, also are important factors interacting with the GM of body [8, 9]. Qinghai-Tibet Plateau, as a high-altitude area in China, is well known for its poor environmental characteristics, such as low atmosphere oxygen, cold and dry weather, and high intensity of ultraviolet radiation. Undergoing chronically natural selection for years then acquiring acclimation, the Tibetan generations become the main one of few ethnic groups living well in the high-altitude areas lastingly. By contrast, the Han have a relatively poorer adaption ability to living well in high-altitude areas, and most of them dwell in the middle-altitude and low-altitude areas in China. But, no study has analyzed the difference in the GM, if any, between the Han and Tibetan HPs in China, and whether composition of GM is in any way correlated with living geographical altitude of the hypertensive hosts to mediate the pathophysiology process of elevating blood pressure is currently unknown.

In this study, we identified the composition of the GM from Tibetan HPs (HTH) and Tibetan HIs (HTN), residing at high-altitude (Qinghai-Tibet Plateau: 3600–4500 m) Han HPs (MHH) and Han HIs (MHN), living at middle-altitude (Xining: 2260 m) and Han HPs (LHH) and Han HIs (LHN) from low-altitude (Wuhan: 13 m), by collecting faecal samples and sequencing bacterial V3-V4 region of 16S rRNA gene. The purpose of this study was to define the distinct features of the GM of these populations and correlate them with the pathology of hypertension in mountain areas.

## 2. Materials and Methods

### 2.1. Study Subjects, Sample Collection, and Ethical Standards

All hypertensive subjects of this study were grade-3 HPs, according to the World Health Organization BP classification, defined as systolic BP ≥ 180 mmHg and/or diastolic BP ≥ 110 mmHg, for more than 3 years. All age- and sex-matched healthy volunteers were physically and psychologically healthy. Fresh stool samples were collected between May 30, 2018, and May 30, 2019, from 225 individuals. Approximately 5 g stool sample was collected in 10 mL sterile tubes without RNAse from each participant at home or inpatient ward, then immediately stored at −20°C, and within 2 h, and transferred to −80°C until used for DNA extraction. The samples were collected from geographical areas with three different altitudes in China. HPs from HTH (*n* = 38) and HIs from HTN (*n* = 34) were Tibetan herders with grade-3 hypertension and healthy volunteers, normally living in Qinghai-Tibet Plateau (altitude: 3600–4500 m); those from MHH (*n* = 49) and MHN (*n* = 35) were Han people with grade-3 hypertension and healthy volunteers living in Xining (altitude: 2260 m); and those from LHH (*n* = 34) and LHN (*n* = 35) were Han people with grade-3 hypertension and healthy volunteers from Wuhan (altitude: 16 m). All hypertensive participants were administered by the calcium channel blocker, combined with angiotensin-converting enzyme (ACE) inhibitor/angiotensin II receptor blocker to control BP. Salt intake of all participants was about 5 g/day. None of the participants had cancer, gastrointestinal diseases, diabetes, obesity, cardiovascular diseases, heart failure, renal failure, stroke, peripheral artery diseases, or any other acute or chronic inflammatory diseases. None used any antibiotics or probiotics within the past 3 months. None was pregnant or lactating. None of them used any drugs, including aspirin, statins, metformin, insulin, or traditional Chinese medicines. The defecation habits of all participants were normal (1-2 times/day).

All experimental protocols were approved by Ethics Committee of the Qinghai University, Xining, China (Ethical approval number: P-SL-2017022 and P-SL-2017063). All methods were carried out in accordance with relevant guidelines. Written informed consents were obtained from all participants.

### 2.2. DNA Extraction

Total genomic DNA from the bacterial community of each stool sample was extracted using the E.Z.N.A. soil DNA kit (M5635-02, Omega, Norcross, Georgia, USA), following the manufacturer's instructions. Concentration of the extracted DNA was estimated, and its quality was determined using a Qubit 3.0 fluorometer (Q10212, Life Technologies, Carlsbad, California, USA).

### 2.3. 16S rRNA Gene Amplification

The V3-V4 hypervariable region of the bacterial 16S rRNA gene was amplified using Taq Master Mix (2×) (P111-03, Vazyme, Nanjing, Jiangsu, China). Two universal bacterial 16S rRNA gene amplicon PCR primers were used: 341F: CCTACGGGNGGCWGCAG and 805R: GACTACHVGGGTATCTAATCC. Unique barcodes were tagged to the 5′-end of the 341F primer to split the sequences of each sample. PCR amplification was performed in duplicate. For each sample, 30 *μ*L reaction mixes were prepared that contained 15 *μ*L 2× Taq master mix, 1 *μ*L bar-PCR primer F (10 *μ*M), 1 *μ*L primer R (10 *μ*M), 10–20 ng genomic DNA, and H_2_O. PCR was carried out using T100TM thermal cycler (BIO-RAD, USA) using the following program: after an initial denaturation step at 94°C for 3 minutes, the targeted region was amplified first 5 cycles of 94*°*C for 30 seconds (s), 45°C for 20 s, and 65°C for 30 s, then 20 cycles of 94°C for 20 s, 55°C for 20 s, and 72°C for 30 s, followed by a final elongation step for 5 minutes at 72°C. PCR products were purified using the Agencourt AMPure XP beads (Beckman Coulter, USA) and were sequenced using Illumina Miseq™ (Truseq Series, Illumina, USA).

### 2.4. Bioinformatics Analysis

The 16S rRNA gene sequences were analyzed and processed using Quantitative Insights Into Microbial Ecology (QIIME v.1.8.0) [10]. It distinguishes sample sequences by their unique barcodes and does quality control for each sample sequence. Subsequently, the barcodes and primer sequences were removed, and then the paired-end sequences were merged with FLASH (v.1.2.3) [11]. USEARCH (v.11.0.667) was used to remove the sequence of the nonamplified region from the preprocessed sequence, and then error correction was made for the sequence [12]. UCHIME (v.4.2.40) was used to identify the chimera [13]. The blastn was used to find the sequence for removed chimera using the representative sequences from the database [14]. Finally, all the remaining sequences were clustered into OTUs at a 97% sequence similarity. The most abundant sequence of each OTU was selected as a representative sequence using USEARCH. Then, the OTUs were aligned to the SILVA database [15], and the OTU table was generated using USEARCH for the subsequent analysis.

The Venn diagram was used to count the shared and unique number of OTUs among different groups and to visually show the similarity and overlap of OTUs among groups. It was drawn using the Venn diagram package (v.1.6.20) of the *R* statistical software (v.3.6.0). The *α*-diversity (Shannon index) and rarefaction curves were calculated using mothur program (v.1.43.0) and plotted in the *R* studio. For the *β*-diversity analysis, the UniFrac distance matrices were calculated using mothur and visualised by principal co-ordinates analysis (PCoA) within the *R* studio. One-way ANOVA and the *t* test were used to test the significance of differences in gastrointestinal microbial composition among different groups and between two groups, respectively. Further, the probability (*p*) < 0.05 was considered statistically significant. Linear discriminant analysis effect size (LEfSe) was used to find the genetic or functional characteristics of the most interpretable differences among different groups and the extent to which these characteristics affect the differences among different groups, which was drawn using the LEfSe software (v.1.1.0). The network diagram was used to show the different abundance of bacteria among different groups by area of the node, which was drawn using the Igraph package (v.1.0.1) in the *R* studio. Bacterial abundance above 1% was analyzed.

## 3. Results

### 3.1. Participant Characteristics and Dietary Information

General characteristics, BP on admission, blood lipid levels, and information about dietary were noted for each participant. Age, gender, body mass index (BMI), smoking status, levels of total cholesterol (TC), triglycerides (TG), low-density lipoprotein cholesterol (LDL-C), high-density lipoprotein cholesterol (HDL-C) as well of systolic BP (SBP), and diastolic BP (DBP), on admission, showed no significant differences among the subgroups of HPs and HIs, *p* > 0.05 (Tables 1 and 2). However, the dietary configurations of different altitude subgroups were quite different. Refined rice, wheat with bran, and zanba (mainly with hulless barley) were the main food of participants in Wuhan, Xining, and Qinghai-Tibet Plateau subgroups, respectively. From low-altitude to high-altitude subgroups, fibre content in the staple food was increasingly higher [16].

### 3.2. Multivariate Statistical Analysis of Gut Community Composition and Diversity Based on OTUs among Different Subgroups according to the Altitude of Living and Ethnicity of HPs and HIs

**Figure 1:**
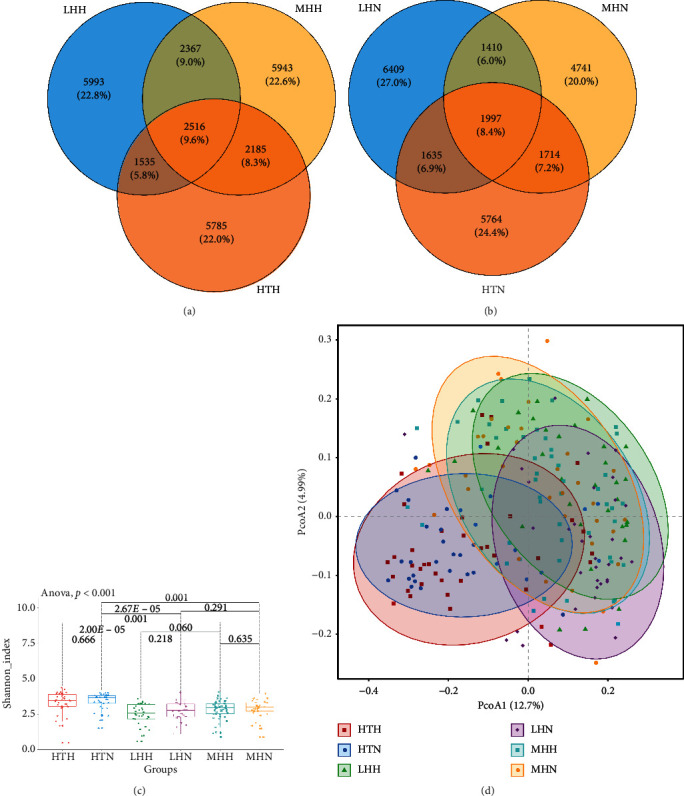
Shared and unique OTUs and *α*-diversity and *β*-diversity of GM among HP subgroups and HI subgroups. Venn diagrams (a) and (b) visually show the similarity and overlap of OTUs. Boxplot (c) was analyzed using Shannon diversity index. The short horizontal line of box from top to bottom stands for 75th, 50th, and 25th percentile, respectively. The *p* value close to vertical axis denotes the significance of differences in GM composition among three subgroups by one-way ANOVA, and other values attached to horizontal lines are *p* values between two subgroups compared by the *t* test in pairs. PCoA chart (d) shows the bidimensional scatter plots of PcoA1 and PcoA2 axes and ellipse represents core position for each group.

Bacterial community profiles were generated for all samples through MiSeq sequencing of 16S rRNA gene. A total of 14,687,874 high-quality sequences were obtained from 225 feces samples. After filtering, we obtained 13,721,382 high-quality sequences (average, 60,984; ranging between 32,622 and 140,217 reads per sample). Then, each sample was rarefied to 24,447 sequences for the evaluation of GM diversity and composition. A total of 49,994 unique operational taxonomic units (OTUs) were detected using UCLSUT algorithm. The Shannon rarefaction curves reached plateau, indicating that sequencing depth of each sample was reasonable and sufficient (Supplementary Figures S1A and S1B). The Venn diagram was used to compare the common and unique OTUs among HPs or HIs subgroups. Of the total 26,324 OTUs were shown in HPs subgroups. In detail, besides about 9.6% (2,516) OTUs shared among LHH, MHH, and HTH, there were about 9.0% (2,367) OTUs shared between LHH and MHH subgroups, about 8.3% (2,185) OTUs were shared between MHH and HTH subgroups, whereas about 5.8% (1535) OTUs were shared only between LHH and HTH subgroups (Figure 1(a)). Among HI subgroups, there were no significant differences in sharing OTUs between paired subgroups (Figure 1(b)).

To evaluate the effects of altitude and ethnicity on the diversity of GM, *α*-diversity (Shannon index) was measured, which revealed a significantly different level of GM diversity among LHH, MHH, HTH, LHN, MHN, and HTN subgroups (Figure 1(c), *p* < 0.001). Within the range of three altitude, the HP subgroup held less *α*-diversity of GM than that the HI subgroup did in the same altitude and ethnicity. (HTH vs. HTN, *p*=0.666; MHH vs. MHN, *p*=0.635; LHH vs. LHN, *p*=0.218, respectively, Figure 1(c)). Meanwhile, among different altitudes and ethnicity subsets of HPs, the diversity of GM in the LHH subgroup was less than that both of the MHH and HTH subgroups (*p*=0.060 and 2.00*E* − 05, respectively, Figure 1(c)), and the HTH subgroup showed the highest diversity of GM. However, the *α*-diversity of GM among HI subgroups showed similar trends but smaller than that among HP subgroups (Figure 1(c)). Next, *β*-diversity measurement of GM was compared among the different altitudes and ethnicity subsets. PCoA based on UniFrac distance matrices took on six distinct enterotypes in the community composition and structure of GM among LHH, MHH, HTH, LHN, MHN, and HTN subgroups (Figure 1(d)).

### 3.3. Taxonomy-Based Comparisons of GM among Different Subgroups according to the Altitude of Living and Ethnicity of HPs and HIs

**Figure 2:**
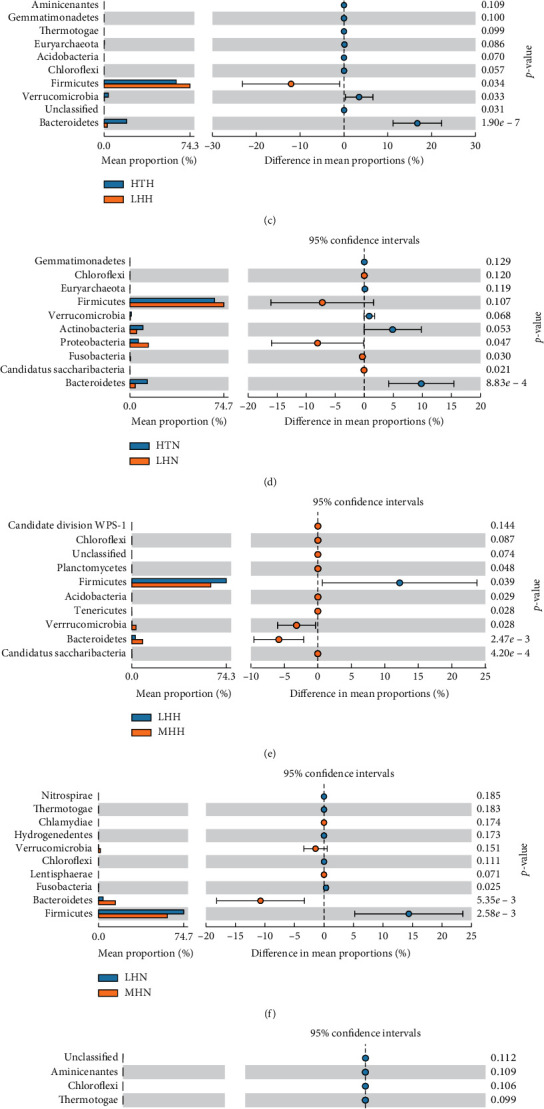
Phylum-based comparisons of GM among subgroups of HPs and HIs. Bar plot (a) displays the proportion of the first six dominant phyla of GM, and different phyla among subgroups are shown in different colors. Scatter diagram (b) presents the different F/B ratio among subgroups. Data are shown as mean ± SD. Error bar plots exhibit different phyla, whose *p* value lies in the bottom ten compared by the *t* test in pairs between two subgroups ((c) HTH vs. LHH; (d) HTN vs. LHN; (e) LHH vs. MHH; (f) LHN vs. MHN; (g) HTH vs. MHH; (h) HTN vs. MHN). The left column shows the mean proportions of each phyla, the middle column shows 95% confidence intervals of the mean proportion of corresponding phyla, and left column lists the corresponding *p* value of each phyla.

First, the mean relative abundance of each phyla and genus was compared among different subgroups by one-way ANOVA (Supplementary Tables S1 and S2). Across all samples, the GM was dominated by Firmicutes, Proteobacteria, Bacteroidetes, Actinobacteria, Verrucomicrobia, and Fusobacteria in both HP and HI groups (Figure 2(a)). As two main kinds of the phylum, the ratio of Firmicutes and Bacteroidetes is used usually to evaluate quantitatively the GM status of the body (Figure 2(b)). Between the HP and HI subgroups from the same altitude and ethnicity, the F/B ratio in HP subgroups was higher than that in the HI subgroups (HTH vs. HTN, *p*=0.410; MHH vs. MHN, *p*=0.373; LHH vs. LHN, *p*=0.075, respectively, Figure 2(b)). Among the different altitudes and ethnicity subsets of HPs and HIs, the F/B ratio was the highest in the LHH subgroup and the lowest in the HTH subgroup as well as HI subgroups showed the similar but smaller changes (LHH vs. HTH, *p*=0.006; LHH vs. MHH, *p*=0.033; and MHH vs. HTH, *p*=0.089; LHN vs. HTN, *p*=0.004; LHN vs. MHN, *p*=0.066; and MHN vs. HTN, *p*=0.073; respectively, Figure 2(b)). Further, we analyzed the differences in the abundance of the GM communities in different subgroups by comparing at the phylum level in pairs by the *t* test. We found the proportion of Firmicutes in both HTH and MHH subgroups was less than that in the LHH subgroup (*p*=0.034 and 0.039, respectively, Figures 2(c) and 2(e)), while that of Bacteroidetes was the highest in the HTH subgroup and the lowest in the LHH subgroup (LHH vs. HTH, *p*=1.90*e* − 7; LHH vs. MHH, *p*=2.47*e* − 3; and MHH vs. HTH, *p*=5.51*e* − 4, respectively, Figures 2(c), 2(e), and 2(g)). Meanwhile, compared to LHH subgroup, the proportion of Verrucomicrobia was higher in both HTH and MHH subgroups (*p*=0.028 and *p*=0.033, respectively, Figures 2(c) and 2(e)). While the abundance of Firmicutes and Bacteroidetes also showed the similar changes but still smaller differences among HI subgroups when compared at the phylum level in pairs by the *t* test (Figures 2(d), 2(f) and 2(h)). In addition, the abundance of Verrucomicrobia showed no significant differences among HI subgroups (Figures 2(d), 2(f), and 2(h)).

**Figure 3:**
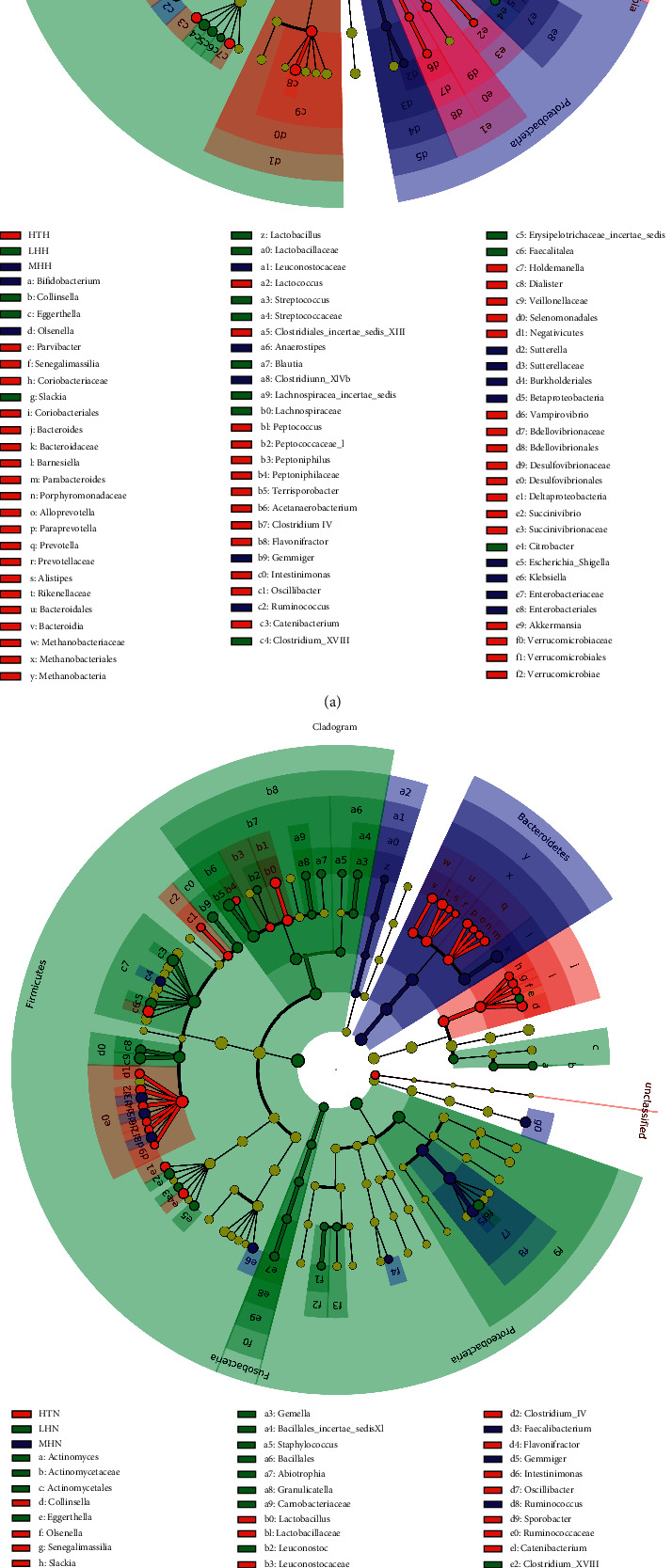
LEfSe analysis of GM composition among HP subgroups (a) and HI subgroups (b). The enriched bacterial taxa in subgroups were represented in cladogram in different colors. The circle radiating from inside to outside represents bacterial taxonomic classification from the phyla to the species, and each node represents one bacterial taxon with proportional diameter representing the relative abundance of the taxon. Alphabetic abbreviations of the corresponding bacteria taxa are defined by column in the right.

The LEfSe results of HP subgroups also showed that most Firmicute bacteria were dominant in the LHH subgroup, while most Bacteroidetes were significantly more abundant in the HTH subgroup (Figure 3(a) and Supplementary Figure S2A) but which were not shown among HI subgroups (Figure 3(b) and Supplementary Figure S2B).

**Figure 4:**
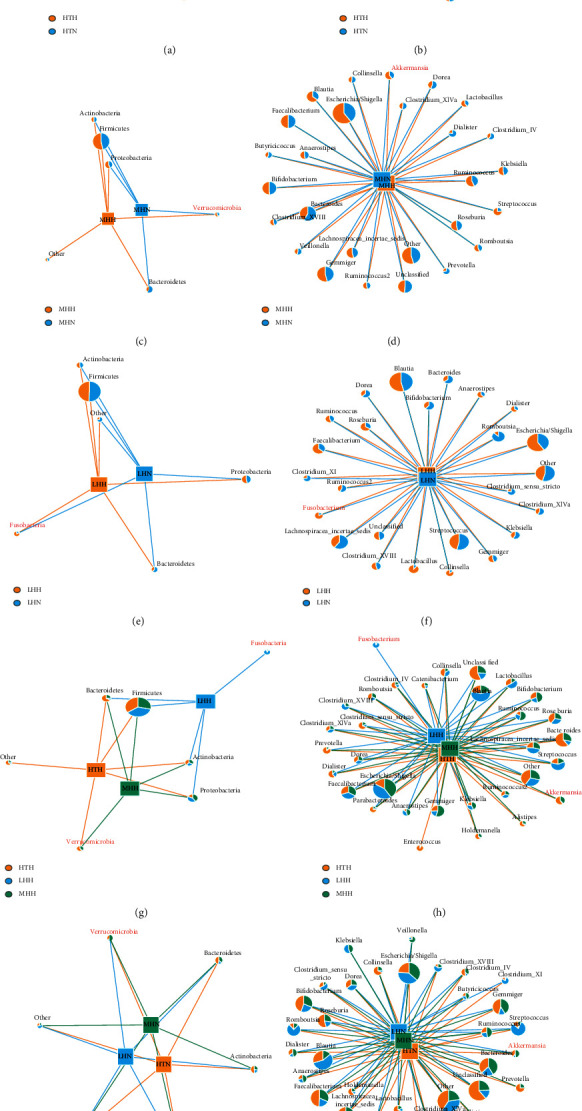
Network diagrams visually show the abundance of taxonomy-based bacteria taxa among subgroups of HPs and HIs ((a), (c), (e), (g), and (i) at phylum level; (b), (d), (f), (h), and (j) at genus level). Different colors represent different groups, different nodes represent different bacterial taxa, and the area of different colors in the nodes represents the abundance of bacterial taxa among subgroups. Bacterial taxa with the abundance greater than 1% were selected to analyse. When drawing, select nodes with significant connections (weight ≥ 100) to draw.

Additionally, between the HP and HI subgroups from the same altitude and ethnicity, the abundance of Verrucomicrobia and its main genera member, Akkermansia, in HPs contributed significantly to the GM of the HTH and MHH subgroups (weight ≥ 100) but hardly connected with the LHH subgroup (weight < 100) at the phylum and genus levels, as per the network results (Figures 4(a)–4(f)), and there also were the same results among HP subgroups (Figures 4(g) and 4(h)), while Fusobacteria and its main component, *Fusobacterium*, were almost exclusively connected with the LHH subgroup (weight ≥ 100), as compared to the LHN subgroup and the other two subgroups of HPs (weight < 100) (Figures 4(e)–4(h)). But, the network results of HI subgroups showed that both Verrucomicrobia and Akkermansia were connected with HTN, MHN, and LHN subgroups with no significant differences at the phylum and genus levels, respectively (weight ≥ 100, Figures 4(i) and 4(g)).

Taken together, taxonomy-based comparisons showed that the GM of the HTH and MHH subgroups had lower F/B ratios and higher abundance of Verrucomicrobia and Akkermansia, compared to that of the HI subgroups from the same altitude and LHH subgroup, which were not totally shown in HI subgroups.

## 4. Discussion

The elevation of BP was related to the undesirable GM composition characterised by less diversity, lower abundance of the phylum Bacteroidetes, genus Akkermansia, and higher F/B ratio, compared to HIs [4, 6]. The GM among the healthy Tibetans from high altitudes was quite different from that of the healthy Han people living at low altitude [17]. In this study, we focused on the distinct features of GM in hypertensive and healthy populations from different altitudes, i.e., at 13 m, 2260 m, and 3600–4500 m, as well as from different ethnic groups, the Han and the Tibetan, respectively. Current studies suggested that the decreasing of GM diversity was a sign of GM dysbiosis [18], and the healthier the body was, the higher the diversity of the GM was [6]. In our study, the diversity of the GM among HPs was lower than that in corresponding HIs from the same altitude and ethnicity, which was in accordance with many research findings related to the relationship between hypertension and the GM [4, 6]. By comparing among HP subgroup, we found the GM of both MHH and HTH subgroups had a higher diversity than that of the LHH subgroup, and the HTH subgroup had the highest GM diversity than the MHH and LHH subgroups did. Moreover, GM distribution among subgroups of HPs and HIs was notably distinct. We speculate that reasons for these, at least to some extent, are mightly related to the following unique dietary habit and external living environmental characteristics of the hosts from plateau areas. Following are some of the reasons which probably are important for the greater diversity of the GM. (1) Higher fibre intake. Dietary fibre was increasingly higher from low-altitude to high-altitude subgroups. And, the studies revealed that high fibre intake had critical influences on the composition of the GM and also was positively correlated the diversity of the GM [19, 20]. (2) Lower temperature and greater hypoxia. It is known that ambient temperature and the partial atmospheric pressure of oxygen gradually decrease as the altitude height increases. For one thing, researches have reported that the GM composition of mammals would change to balance their energy homeostasis in the condition of the cold environment [21, 22], and diversity of GM was shown to decrease when environmental temperature rose [23, 24]. For another, Hypoxia may be another factor that influences the diversity and composition of the GM [25]. Moreno-Indias et al. [26] reported that the diversity in the GM was much richer in individuals living in low-oxygen conditions than those from normal oxygen conditions. (3) Lower degree of industrialisation and urbanization. In this study, participants of high-altitude came from unindustrialised rural areas, those of middle-altitude from developing industrialized urban areas, whereas individuals of low-altitude from developing industrialized urban areas. It was demonstrated that the diversity of GM was related to the degree of industrialisation and urbanization [27, 28]. Probably, the reasons were that unindustrialised rural communities used less antibiotics and cleaners and ate much healthier foods, which were good for GM. In summary, high-fibre diet, cold climate, hypoxic conditions, and low industrialisation urbanization commonly or separately might be some factors contributing to the high diversity and distinct composition of GM in HPs living at high altitudes.

However, the differences of GM distribution and composition among HI subgroups were much smaller than those differences in HP subgroups. So, we speculated that different living environments are not the only factors for specific features of GM in high-altitude Tibetan and middle-altitude Han HPs.

The human intestine is inhabited by about 100 trillion gut bacteria [29]. Generally, they can be either beneficial or pathogenic organisms. Firmicutes and Bacteroidetes are two dominant phyla in the intestine, with variable proportions under different disease states, and mostly, the former was pathogenic [30] but the latter was beneficial to host health [18]. So, F/B ratio was gradually considered as a potential biomarker for various pathological statuses [30], and high F/B ratio was reported to be closely associated with obesity, diabetes mellitus, and hypertension [31–33]. However, we found the proportion of Firmicutes was less in the HTH subgroup and that of Bacteroidetes was significantly more in MHH and HTH subgroups, compared to that in the LHH subgroup. As a result, the F/B ratio decreased with altitude increasing. But, the results of HI subgroups showed smaller differences in the F/B ratio. It has been shown that exposure to high-altitude could alter the composition of GM [34]. And, the Tibetans and the Hans had several differences in genotype frequencies [35], which can affect on the composition of the GM [36]. Therefore, genetic and geographical altitude factors may explain these differences in the composition of GM among HP subgroups or HI subgroup. But, the differences of GM distribution and composition among HI subgroups were much smaller than those in HP subgroups. This reflected the GM of individuals had different adaptations to the plateau environment under hypertensive or healthy conditions. It is possible that these two factors may have greater impacts on the GM in HPs, and unique GM characteristics may take roles in regulating the BP of HPs in plateau.

Additionally, according to our data, the abundance of both Verrucomicrobia and Akkermansia was higher in both MHH and HTH subgroups than that in the LHH subgroup and corresponding HIs subgroups from the same altitude and ethnicity. Fusobacteria, such as Fusobacterium, were the most common bacterium in the LHH subgroup but not so in the other two HP subgroups and LHN subgroup. And, these results showed no significant differences among HI subgroups. The GM was linked to a diverse range of conditions, including gathering energy and enhancing the immune system [37]. Verrucomicrobia, as one of the common phyla of human GM, was involved in the process of host energy metabolism by degrading polysaccharides of the fermentations of the intestinal tract [38, 39]. Akkermansia, the most important member of this taxon, has attracted largely extensive attention owing to its famous beneficial and protective effects on the host [40], such as weight loss, lipid lowering, and antiatherosclerosis by suppressing systemic inflammatory response [41–43], whereas Fusobacteria were associated with periodontal diseases and colorectal cancer by activating inflammatory responses [44, 45]. Furthermore, the positive relationship between hypertension and systemic inflammation has been consistently confirmed by many researchers [46]. When the blood vessels are stretched under the condition of hypertension, endothelium will be stimulated to release proinflammatory cytokines. Further, they can stimulate and activate surrounding monocytes, macrophages, DCs, and T lymphocytes and aggravate the secretion process of these proinflammatory cytokines, which take part in the pathological process of hypertension [47]. So, compared to the HIs from the same altitude and ethnicity, Verrucomicrobia and Akkermansia might be good for high-altitude Tibetan and middle-altitude Han HPs by controlling the inflammatory response and balancing the energy metabolism, whereas highly expressed Fusobacteria and Fusobacterium might thrive in the inflammatory reaction to aggravate the hypertensive pathogenesis of low-altitude Han HPs. However, because of regional differences of population distribution, it was quite difficult to recruit Tibetan HPs settled in Wuhan or Xining and Han HPs settled in Qinghai-Tibet Plateau. Therefore, we could not independently analyse the impact of ethnicity on the GM diversity and distribution among HPs in the plateau.

## 5. Conclusions

This study showed that the beneficial changes of GM in HTH and MHH subgroups, including greater *α*- and *β*-diversity, lower ratio of F/B, and higher abundance of Verrucomicrobia and Akkermansia, which were not totally shown in HTN and MHN subgroups of HI group and the LHH subgroup. Such specific features of the GM might play protective roles in regulating BP of high-altitude Tibetans and middle-altitude Hans. Next, we plan to study the role played by the gut bacteria in acquiring specific adaptations needed to regulate BP in the plateau environment, using in vivo and in vitro models and elucidate the precise underlying mechanisms.

## Figures and Tables

**Table 1:** Baseline clinical characteristics of participants among subgroups of HPs.

Baseline clinical characteristics	LHH (*n* = 34)	MHH (*n* = 49)	HTH (*n* = 38)	*p* value
Age (years)	55.9 ± 9.7	55.1 ± 8.7	52.8 ± 10.6	0.349
Sex (male/female)	16/18	28/21	23/15	0.491
Smoking, *n* (%)	15 (44.11)	27 (55.10)	16 (52.63)	0.467
BMI (kg/m^2^)	23.94 ± 0.60	23.64 ± 1.03	24.01 ± 0.94	0.137
SBP (mmHg)	147.7 ± 14.1	146.7 ± 10.2	146.6 ± 15.8	0.934
DBP (mmHg)	84.7 ± 15.4	82.6 ± 6.4	83.4 ± 11.2	0.693
TC (mmol/L)	4.03 ± 0.82	4.24 ± 0.78	4.16 ± 0.86	0.540
TG (mmol/L)	1.06 ± 0.11	1.07 ± 0.12	1.06 ± 0.12	0.797
HDL-C (mmol/L)	1.14 ± 0.33	1.03 ± 0.24	1.04 ± 0.27	0.208
LDL-C (mmol/L)	2.38 ± 0.63	2.53 ± 0.85	2.73 ± 0.68	0.135

Values are expressed as mean ± SD or *n* (%). *p* values are calculated by one-way ANOVA or chi-squared test, and *p* < 0.05 was considered statistically significant. Abbreviations: LHH, the Han HPs from low-altitude (Wuhan: 13 m); MHH, the Han HPs living at middle-altitude (Xining: 2260 m); HTH, the Tibetan HPs residing at high-altitude (Qinghai-Tibet Plateau: 3600–4500 m); BMI, Body Mass Index; SBP, systolic blood pressure; DBP, diastolic blood pressure; TC, total cholesterol; TG, triglyceride; HDL-C, high-density lipoprotein cholesterol; LDL-C, low-density lipoprotein cholesterol.

**Table 2:** Baseline clinical characteristics of participants among subgroups of HIs.

Baseline clinical characteristics	LHN (*n* = 35)	MHN (*n* = 35)	HTN (*n* = 34)	*p* value
Age (years)	52.1 ± 6.9	51.9 ± 7.6	53.2 ± 9.3	0.778
Sex (male/female)	21/14	15/20	18/16	0.353
Smoking, *n* (%)	14 (42.11)	14 (40.00)	18 (52.94)	0.460
BMI (kg/m^2^)	23.64 ± 1.12	23.48 ± 1.13	24.00 ± 1.00	0.137
SBP (mmHg)	118.9 ± 3.2	118.0 ± 4.1	119.2 ± 3.8	0.386
DBP (mmHg)	79.1 ± 2.8	78.6 ± 3.6	76.7 ± 6.4	0.063
TC (mmol/L)	4.18 ± 0.55	4.35 ± 1.13	4.26 ± 1.02	0.755
TG (mmol/L)	1.08 ± 0.17	1.11 ± 0.13	1.05 ± 0.14	0.183
HDL-C (mmol/L)	1.10 ± 0.15	1.12 ± 0.29	1.02 ± 0.22	0.140
LDL-C (mmol/L)	2.54 ± 0.90	2.67 ± 0.87	2.83 ± 0.93	0.392

Abbreviations: LHN, the Han HIs from low-altitude (Wuhan: 13 m); MHN, the Han HIs living at middle-altitude (Xining: 2260 m); HTN, the Tibetan HIs residing at high-altitude (Qinghai-Tibet Plateau: 3600–4500 m); statistical parameters and the other abbreviations as in Table 1.

## Data Availability

The data used to support the study are available within the article.

## Supplementary Materials

Supplementary Materials Supplementary Figures S1 and S2 and Supplementary Tables S1 and S2 in the Supplementary Material for comprehensive image analysis.
