# Supplementary material for: Distinct Features of Gut Microbiota in High-Altitude Tibetan and Middle-Altitude Han Hypertensive Patients
Source: Cardiol Res Pract. 2020 Nov 21;2020:1957843. doi: 10.1155/2020/1957843 (PMC7700061; doi:10.1155/2020/1957843)
Supplement: Supplementary Materials — Supplementary Figures S1 and S2 and Supplementary Tables S1 and S2 in the Supplementary Material for comprehensive image analysis. [file 1957843.f1.zip › Supplemental Files/Supplementary Figure S1A.pdf]

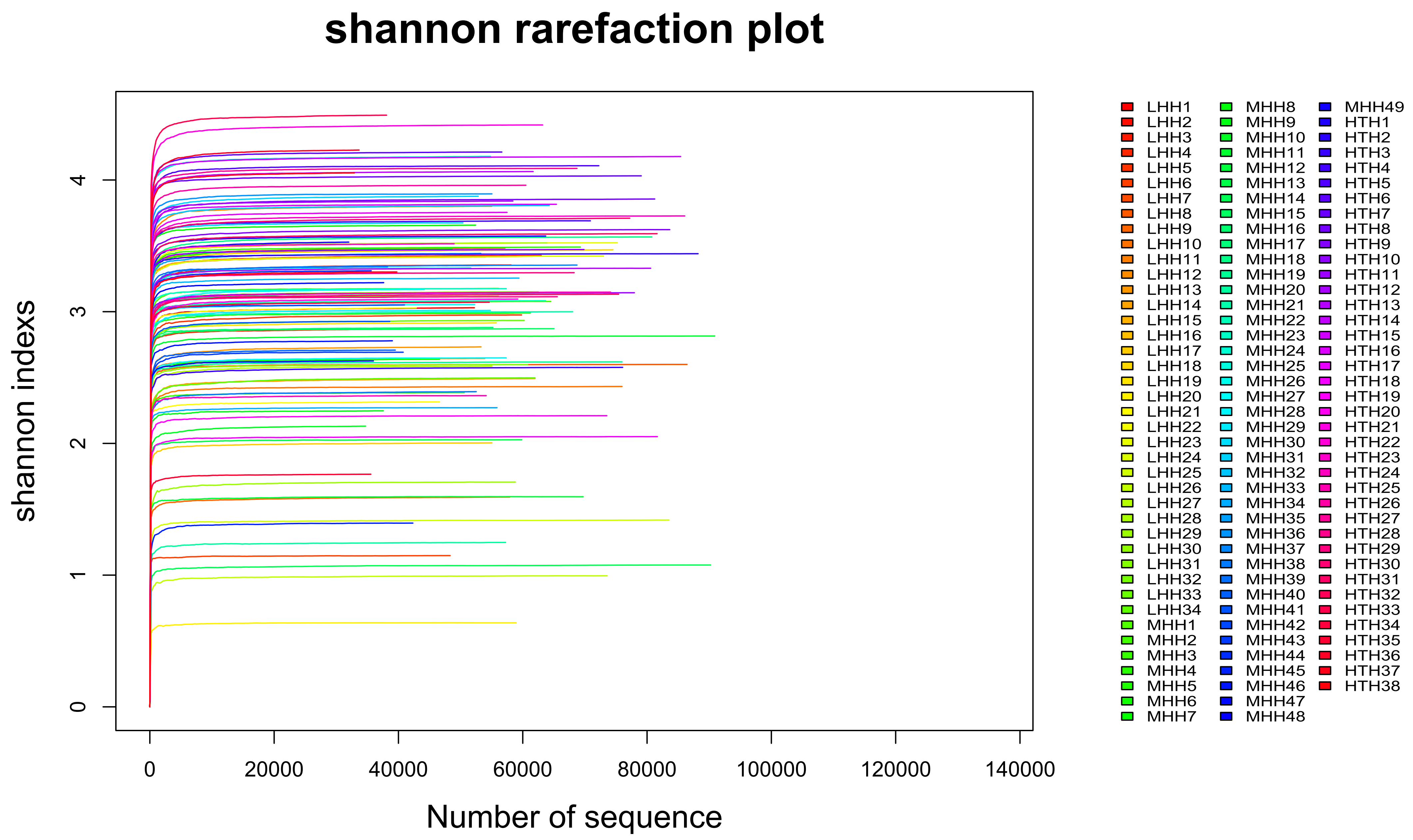
shannon rarefaction plot
shannon indexs
0
1
2
3
4
Number of sequence
0
20000
40000
60000
80000
100000
120000
140000
LHH1 LHH2 LHH3 LHH4 LHH5 LHH6 LHH7 LHH8 LHH9 LHH10 LHH11 LHH12 LHH13 LHH14 LHH15 LHH16 LHH17 LHH18 LHH19 LHH20 LHH21 LHH22 LHH23 LHH24 LHH25 LHH26 LHH27 LHH28 LHH29 LHH30 LHH31 LHH32 LHH33 LHH34
MHH1 MHH2 MHH3 MHH4 MHH5 MHH6 MHH7 MHH8 MHH9 MHH10 MHH11 MHH12 MHH13 MHH14 MHH15 MHH16 MHH17 MHH18 MHH19 MHH20 MHH21 MHH22 MHH23 MHH24 MHH25 MHH26 MHH27 MHH28 MHH29 MHH30 MHH31 MHH32 MHH33 MHH34 MHH35 MHH36 MHH37 MHH38 MHH39 MHH40 MHH41 MHH42 MHH43 MHH44 MHH45 MHH46 MHH47 MHH48 MHH49
HTH1 HTH2 HTH3 HTH4 HTH5 HTH6 HTH7 HTH8 HTH9 HTH10 HTH11 HTH12 HTH13 HTH14 HTH15 HTH16 HTH17 HTH18 HTH19 HTH20 HTH21 HTH22 HTH23 HTH24 HTH25 HTH26 HTH27 HTH28 HTH29 HTH30 HTH31 HTH32 HTH33 HTH34 HTH35 HTH36 HTH37 HTH38
