# Supplementary figures and images for: Distinct Features of Gut Microbiota in High-Altitude Tibetan and Middle-Altitude Han Hypertensive Patients
Source: Cardiol Res Pract. 2020 Nov 21;2020:1957843. doi: 10.1155/2020/1957843 (PMC7700061; doi:10.1155/2020/1957843)

# shannon rarefaction plot

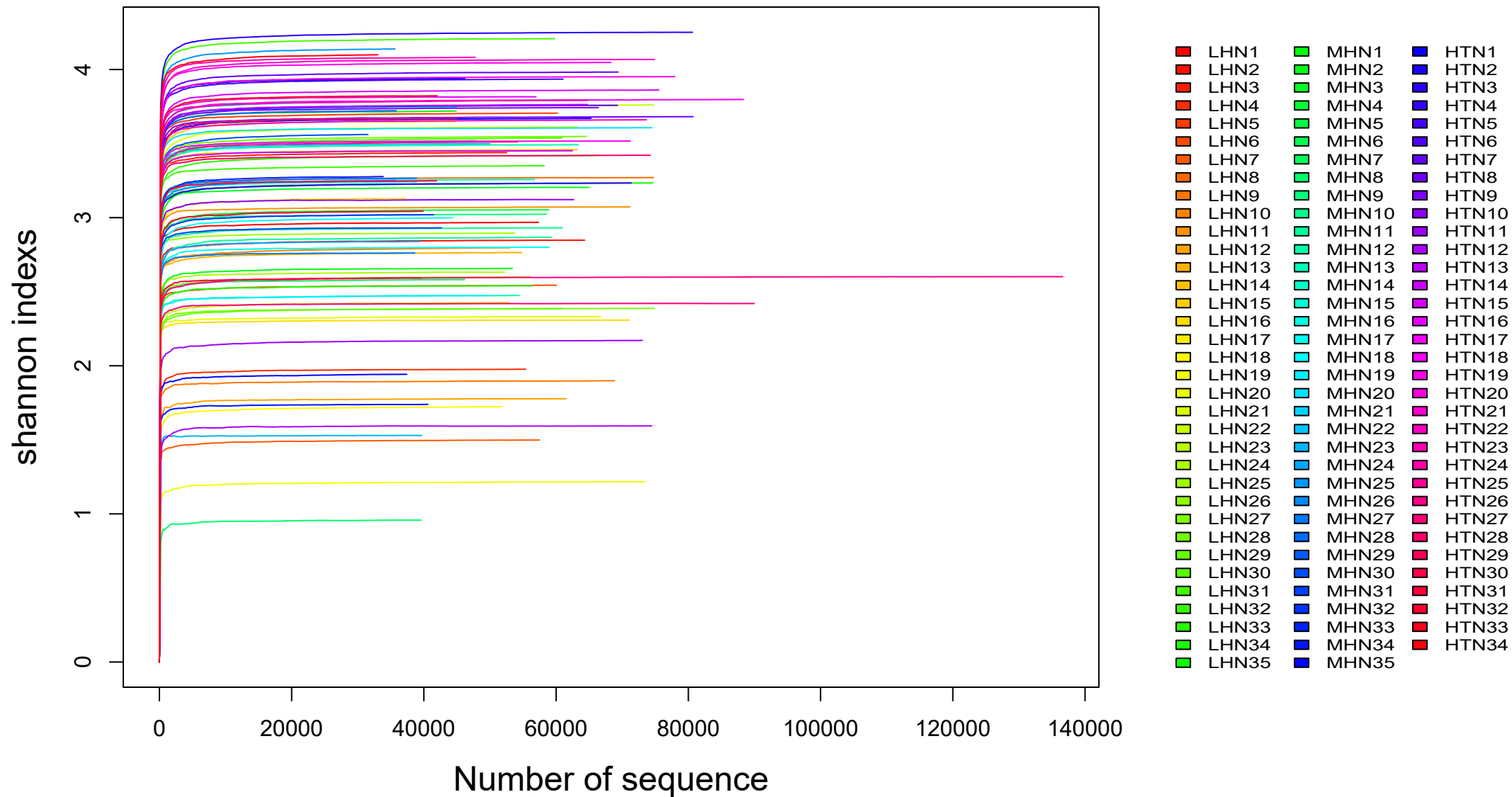

Supplement: Supplementary Materials — Supplementary Figures S1 and S2 and Supplementary Tables S1 and S2 in the Supplementary Material for comprehensive image analysis. [file 1957843.f1.zip › Supplemental Files/Supplementary Figure S1B.pdf]

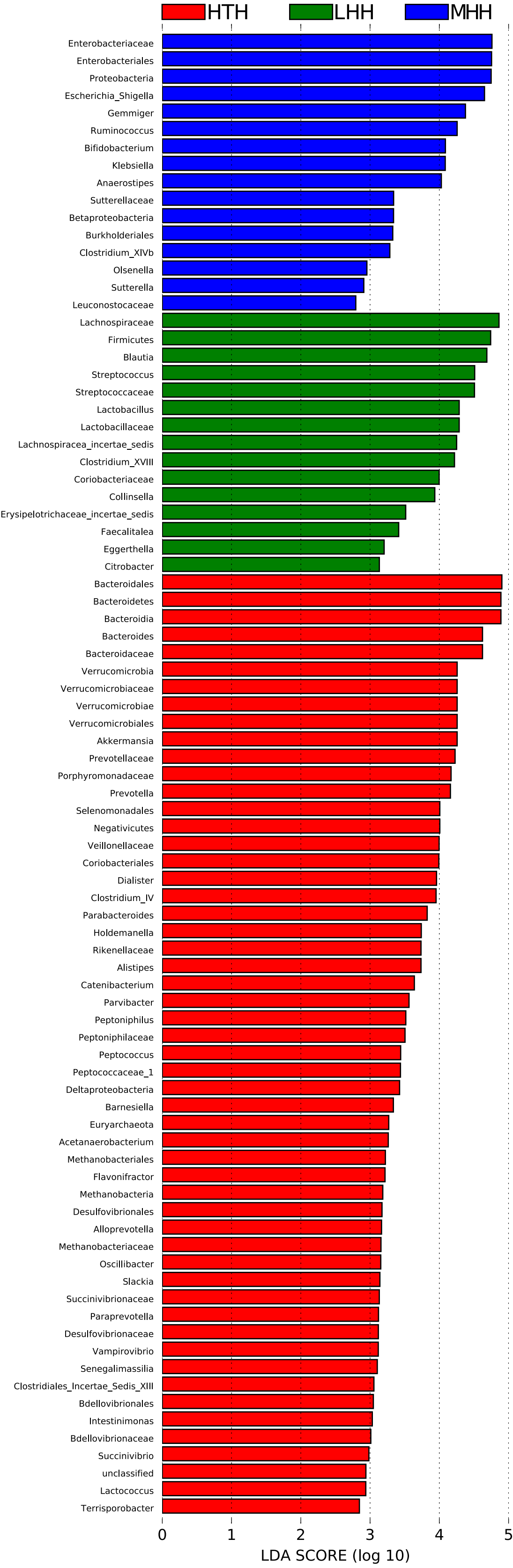

Supplement: Supplementary Materials — Supplementary Figures S1 and S2 and Supplementary Tables S1 and S2 in the Supplementary Material for comprehensive image analysis. [file 1957843.f1.zip › Supplemental Files/Supplementary Figure S2A.pdf]

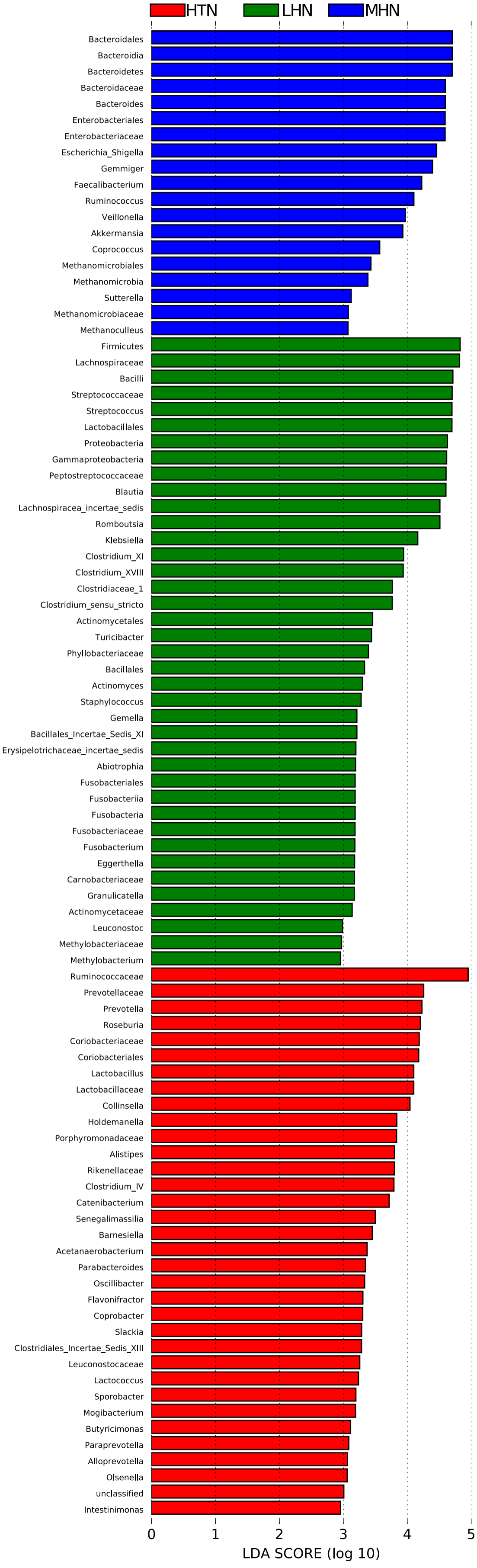

Supplement: Supplementary Materials — Supplementary Figures S1 and S2 and Supplementary Tables S1 and S2 in the Supplementary Material for comprehensive image analysis. [file 1957843.f1.zip › Supplemental Files/Supplementary Figure S2B.pdf]
