# Supplementary material for: Distinct Features of Gut Microbiota in High-Altitude Tibetan and Middle-Altitude Han Hypertensive Patients
Source: Cardiol Res Pract. 2020 Nov 21;2020:1957843. doi: 10.1155/2020/1957843 (PMC7700061; doi:10.1155/2020/1957843)
Supplement: Supplementary Materials — Supplementary Figures S1 and S2 and Supplementary Tables S1 and S2 in the Supplementary Material for comprehensive image analysis. [file 1957843.f1.zip › Supplemental Files/Supplementary Materials Legends.doc]

**Supplementary Materials Legends:**

**Supplementary Figure S1:** Shannon rarefaction curves based on OTUs among HPs sub-groups (A) and HIs sub-groups (B). It was detected using a similarity threshold of 97%. Rarefaction curve of each sample is shown in different color.

**Supplementary Figure S2:** The linear discriminant analysis of GM composition among HPs sub-groups (A) and HIs sub-groups (B). The most positively and uniquely enriched abundant taxa are shown through the LDA score (>2).

**Supplementary Table S1:** The List of the Relative Abundance of Each Phyla of GM among Sub-groups of HPs and HIs

**Supplementary Table S2:** The List of the Relative Abundance of Each Phyla of GM among Sub-groups of HPs and HIs
