# Supplementary material for: Distinct Features of Gut Microbiota in High-Altitude Tibetan and Middle-Altitude Han Hypertensive Patients
Source: Cardiol Res Pract. 2020 Nov 21;2020:1957843. doi: 10.1155/2020/1957843 (PMC7700061; doi:10.1155/2020/1957843)
Supplement: Supplementary Materials — Supplementary Figures S1 and S2 and Supplementary Tables S1 and S2 in the Supplementary Material for comprehensive image analysis. [file 1957843.f1.zip › Supplemental Files/Supplementary Table S1.doc]

**Distinct Features of Gut Microbiota in High-altitude Tibetan and Middle-altitude Han Hypertensive Patients**

**Lu-lu Zhu^a,^*, Zhi-jun Ma^b,^*, Ming Ren^a,^*, Yu-miao Wei^c^, Yu-hua Liao^c^, You-lu Shen^a^, Shi-ming Fan^a^, Lin Li^a^, Qing-xia Wu^a^, Zhong-shan Gao^a^, Jing-fu Song^d^, and Yu-lan Ma^a,^****

| Supplementary Table S1 The List of the Relative Abundance of Each Phyla of GM among Sub-groups of HPs and HIs | | | | | | |
| --- | --- | --- | --- | --- | --- | --- |
|  | LHH(n=34) | MHH(n=49) | HTH(n=38) | LHN(n=35) | MHN(n=35) | HTN(n=34) |
| Firmicutes(%) | 74.3141±25.8212 | 62.0994±26.2220 | 62.2529±20.6368 | 74.7494±20.0571 | 60.3794±18.3140 | 67.5211±16.6018 |
| Proteobacteria(%) | 15.0500±26.7245 | 18.6241±23.4776 | 7.1689±10.3275 | 14.7500±17.7042 | 14.4169±18.9303 | 6.7088±15.1959 |
| Bacteroidetes(%) | 2.8074±6.0031 | 8.6412±10.8831 | 19.5424±15.7722 | 4.1751±10.4985 | 14.9251±19.2110 | 13.9876±12.7246 |
| Actinobacteria(%) | 6.3409±9.0729 | 6.9733±8.4660 | 7.1032±7.3049 | 5.41456±9.1816 | 8.2523±10.63144 | 10.2868±11.2307 |
| Verrucomicrobia(%) | 0.1894±0.6451 | 3.3845±9.8482 | 3.6342±9.5842 | 0.3883±1.0763 | 1.8203±5.667 | 1.2418±2.4417 |
| Fusobacteria(%) | 1.2471±7.1604 | 0.1788±0.6944 | 0.0784±0.3733 | 0.3620±0.8660 | 0.0183±0.0350 | 0.0297±0.0769 |
| Euryarchaeota(%) | 0.0268±0.0868 | 0.0149±0.0290 | 0.1405±0.3881 | 0.0397±0.0968 | 0.1075±0.5201 | 0.1435±0.3676 |
| Candidatus Saccharibacteria(%) | 0.0141±0.0183 | 0.0427±0.0484 | 0.0205±0.0307 | 0.0677±0.1102 | 0.0589±0.0985 | 0.0224±0.0361 |
| Unclassified(%) | 0.0029±0.0072 | 0.0088±0.0226 | 0.0276±0.0684 | 0.0177±0.0468 | 0.0066±0.0189 | 0.0347±0.0873 |
| Tenericutes(%) | 0.0003±0.0017 | 0.0167±0.0505 | 0.0024±0.0115 | 0.0006±0.0024 | 0.0017±0.0101 | 0.0024±0.0089 |
| Synergistetes(%) | 0.0024±0.009 | 0.0045±0.0123 | 0.0047±0.0103 | 0.0049±0.0156 | 0.0049±0.0162 | 0.0050±0.0144 |
| Chloroflexi(%) | 0.0003±0.0017 | 0.0012±0.0039 | 0.0076±0.0234 | 0.0063±0.0207 | 0.0008±0.0037 | 0.0008±0.0029 |
| Planctomycetes(%) | 0.0006±0.0034 | 0.0037±0.0091 | 0.0034±0.0101 | 0.0043±0.0150 | 0.0011±0.0053 | 0.0021±0.0054 |
| Acidobacteria(%) | 0.0006±0.0023 | 0.0024±0.0056 | 0.0032±0.0078 | 0.0057±0.0167 | 0.0029±0.0093 | 0.0035±0.0088 |
| Gemmatimonadetes(%) | 0.0003±0.0017 | 0.0006±0.0024 | 0.0018±0.0046 | 0.0006±0.0024 | 0 | 0.0021±0.0054 |
| Lentisphaerae(%) | 0 | 0.0002±0.0014 | 0 | 0 | 0.0003±0.0017 | 0.0024±0.0105 |
| Crenarchaeota(%) | 0.0003±0.0017 | 0 |  | 0.0011±0.0040 | 0.0005±0.0034 | 0.0000±0.0000 |
| Aminicenantes(%) | 0 | 0 | 0.0008±0.0027 | 0.000571±0.0033806 | 0 | 0 |
| Candidate division WPS-1(%) | 0 | 0.0002±0.0014 | 0.0005±0.0023 | 0 | 0 | 0.0003±0.0017 |
| Nitrospirae(%) | 0 | 0 | 0.0003±0.0016 | 0.0011±0.0040 | 0 | 0 |
| Parcubacteria(%) | 0 | 0.0002±0.0014 | 0.0005±0.0023 | 0.0006±0.0034 | 0 | 0 |
| Deinococcus  -Thermus(%) | 0 | 0.0002±0.0014 | 0 | 0.0011±0.0053 | 0 | 0 |
| Thermotogae(%) | 0 | 0 | 0.0003±0.0016 | 0.0009±0.0037 | 0 | 0 |
| Chlamydiae(%) | 0 | 0 | 0.0005±0.0023 | 0 | 0 | 0 |

Values are expressed as mean±SD. Abbreviations: LHH, the Han HPs from low-altitude (Wuhan: 13 m); MHH, the Han HPs living at middle-altitude (Xining: 2260 m); HTH, the Tibetan HPs residing at high-altitude (Qinghai-Tibet Plateau: 3600–4500 m); LHN, the Han HIs from low-altitude (Wuhan: 13 m); MHN, the Han HIs living at middle-altitude (Xining: 2260 m); HTN, the Tibetan HIs residing at high-altitude (Qinghai-Tibet Plateau: 3600–4500 m).
