# Supplementary material for: Distinct Features of Gut Microbiota in High-Altitude Tibetan and Middle-Altitude Han Hypertensive Patients
Source: Cardiol Res Pract. 2020 Nov 21;2020:1957843. doi: 10.1155/2020/1957843 (PMC7700061; doi:10.1155/2020/1957843)
Supplement: Supplementary Materials — Supplementary Figures S1 and S2 and Supplementary Tables S1 and S2 in the Supplementary Material for comprehensive image analysis. [file 1957843.f1.zip › Supplemental Files/Supplementary Table S2.doc]

**Distinct Features of Gut Microbiota in High-altitude Tibetan and Middle-altitude Han Hypertensive Patients**

**Lu-lu Zhu^a,^*, Zhi-jun Ma^b,^*, Ming Ren^a,^*, Yu-miao Wei^c^, Yu-hua Liao^c^, You-lu Shen^a^, Shi-ming Fan^a^, Lin Li^a^, Qing-xia Wu^a^, Zhong-shan Gao^a^, Jing-fu Song^d^, and Yu-lan Ma^a,^****

| Supplementary Table S2 The List of the Relative Abundance of Each Genus of GM among Sub-groups of HPs and HIs | | | | | | |
| --- | --- | --- | --- | --- | --- | --- |
|  | LHH（n=34） | MHH（n=49） | HTH（n=38） | LHN(n=35) | MHN(n=35) | HTN(n=34) |
| *Escherichia/Shigella*  (%) | 11.8497±26.2936 | 13.9233±20.5381 | 5.0042±9.2505 | 9.1923±17.4941 | 10.3034±17.9136 | 5.3347±14.6760 |
| *Blautia*(%) | 13.5932±12.1120 | 5.5767±5.5774 | 4.4421±5.8588 | 11.2169±6.6270 | 3.3949±4.0235 | 5.2612±4.9201 |
| *unclassified*(%) | 3.4888±3.5720 | 5.7188±7.9062 | 10.7989±9.5211 | 3.1983±6.5383 | 6.2823±7.0286 | 12.5479±10.5672 |
| *Bacteroides*(%) | 2.1347±5.1946 | 6.4812±9.7016 | 11.5068±12.6752 | 3.6751±10.1472 | 11.6291±17.2367 | 6.7926±7.3511 |
| *Faecalibacterium*(%) | 5.7653±8.0088 | 5.9322±7.1256 | 4.8800±4.9513 | 3.4249±5.7644 | 7.0583±6.7605 | 6.6624±8.5317 |
| *Gemmiger*(%) | 2.4138±4.1679 | 7.6937±10.6925 | 3.1979±4.1375 | 1.9197±3.7969 | 7.3037±7.0969 | 6.4668±8.3882 |
| *Streptococcus*(%) | 9.4315±13.2301 | 2.8445±5.6996 | 2.7495±6.5197 | 10.3540±13.5806 | 1.0300±1.3777 | 1.60744±2.6767 |
| *Lachnospiracea_*  *incertae_sedis*(%) | 6.5562±6.8546 | 4.3478±7.5829 | 3.0179±3.4046 | 9.6266±9.7423 | 4.6249±11.7564 | 3.1974±4.4766 |
| *Roseburia*(%) | 4.0659±5.0111 | 4.0829±5.8508 | 4.4911±6.1097 | 2.0469±4.4584 | 3.6571±4.3195 | 5.5444±6.4102 |
| *Bifidobacterium*(%) | 2.7071±4.8150 | 5.2743±8.4069 | 3.3689±3.9317 | 4.0120±9.1009 | 6.4920±9.0092 | 6.3641±9.4095 |
| *Lactobacillus*(%) | 5.6456±13.7760 | 1.8969±6.6597 | 3.0747±5.5230 | 0.6397±1.2837 | 1.2469±3.4965 | 3.0282±5.8264 |
| *Ruminococcus*(%) | 2.4306±6.2354 | 4.9820±7.7660 | 1.4805±2.5600 | 1.9217±6.6610 | 4.1503±7.1105 | 1.8165±3.4686 |
| *Akkermansia*(%) | 0.1876±0.6395 | 3.3824±9.8444 | 3.6324±9.5842 | 0.3749±1.0764 | 1.8189±5.6638 | 1.2385±2.4426 |
| *Collinsella*(%) | 2.7494±5.8113 | 1.0894±1.7526 | 2.6789±4.4824 | 0.3786±1.1630 | 1.3489±3.68587 | 2.5459±3.8557 |
| *Klebsiella*(%) | 1.9221±8.6744 | 3.2949±8.8287 | 1.0050±5.6783 | 2.7931±6.7126 | 2.3240±6.9684 | 0.1191±0.4168 |
| *Dorea*(%) | 1.8926±1.8421 | 1.9304±1.9975 | 1.8371±1.8067 | 3.1783±3.5312 | 2.4957±2.8173 | 2.5250±3.0108 |
| *Romboutsia*(%) | 1.3218±2.1621 | 1.8418±2.9136 | 1.8524±4.9210 | 8.4697±13.9198 | 1.5714±3.0390 | 1.2800±2.1698 |
| *Dialister*(%) | 1.3979±6.8004 | 0.8222±2.4861 | 2.5895±7.1505 | 0.6749±2.0399 | 2.3791±6.1728 | 1.8697±4.8043 |
| *Ruminococcus2*(%) | 1.8300±2.3981 | 1.4924±1.7231 | 1.4432±1.1568 | 2.4371±3.0194 | 1.4417±1.5040 | 1.9650±1.5748 |
| *Clostridium sensu stricto*(%) | 0.6256±1.0059 | 0.7961±2.0581 | 2.4947±6.3914 | 2.1429±6.6776 | 0.6126±1.228 | 1.1965±2.2313 |
| *Anaerostipes*(%) | 1.7759±3.1712 | 2.3051±6.0087 | 0.4766±1.0829 | 1.0577±1.2813 | 2.7180±8.4739 | 1.0897±2.4066 |
| *Clostridium XlVa*(%) | 1.7406±3.4523 | 1.2496±1.4836 | 1.4913±2.3853 | 2.2869±5.5583 | 1.5883±3.0316 | 1.2971±1.9401 |
| *Prevotella*(%) | 0.1779±0.5373 | 0.4898±1.1012 | 3.1547±4.5020 | 0.0266±0.0775 | 1.3283±3.1413 | 3.6056±6.1826 |
| *Clostridium XVIII*(%) | 3.3212±12.0909 | 1.0916±1.4124 | 0.2468±0.3939 | 2.4000±2.3544 | 0.9960±1.4067 | 0.5800±0.8499 |
| *Clostridium IV*(%) | 0.7044±1.26.5 | 0.9831±1.9182 | 2.3361±3.4331 | 0.3766±0.7088 | 1.3331±1.9973 | 1.6753±2.2264 |
| *Catenibacterium*(%) | 0.5024±2.9274 | 0.6202±2.2442 | 1.1816±2.1950 | 0.0014±0.0043 | 0.1666±0.6216 | 1.0456±1.8698 |
| *Holdemanella*(%) | 0.1300±0.3867 | 0.7978±3.1463 | 1.1524±1.6237 | 0.3117±1.83729 | 0.6351±1.6406 | 1.4865±3.1132 |
| *Parabacteroides*(%) | 0.2529±0.7968 | 0.4035±1.3365 | 1.5684±2.1370 | 0.3140±0.5897 | 0.8060±1.7007 | 0.8376±1.1799 |
| *Enterococcus*(%) | 0.0615±0.1801 | 0.0976±0.2929 | 2.3221±13.9124 | 0.1274±0.3743 | 0.1337±0.5813 | 0.2503±0.7262 |
| *Alistipes*(%) | 1.1391±0.3015 | 0.6149±1.0176 | 1.2061±1.8519 | 0.0789±0.2476 | 0.5875±0.8719 | 1.4938±2.7004 |
| *Coprococcus*(%) | 0.5341±0.9120 | 0.7071±0.7529 | 0.6261±0.5255 | 0.2200±0.4433 | 0.8840±1.3204 | 0.8659±0.7450 |
| *Fusicatenibacter*(%) | 0.8318±1.4873 | 0.6390±0.9310 | 0.4553±0.5625 | 0.7451±1.0498 | 0.4106±0.4237 | 0.4029±0.5048 |
| *Fusobacterium*(%) | 1.2468±7.1604 | 0.1782±0.6944 | 0.0742±0.3715 | 0.3609±0.8660 | 0.0183±0.0350 | 0.0282±0.0761 |
| *Butyricicoccus*(%) | 0.7415±1.0671 | 0.7092±0.9407 | 0.3571±0.4670 | 0.4751±0.5790 | 0.9600±1.4245 | 0.3953±0.3665 |
| *Clostridium XI*(%) | 0.6476±1.7943 | 0.2524±0.4194 | 0.6239±1.3131 | 1.9386±7.4943 | 0.1306±0.2954 | 0.3541±0.5652 |
| *Veillonella*(%) | 0.3044±1.5290 | 0.8814±3.3966 | 0.4045±1.9428 | 0.2080±0.4997 | 1.4229±6.7932 | 0.1697±0.5403 |
| *Flavonifractor*(%) | 0.2297±0.4010 | 0.2837±0.4399 | 0.5795±0.8739 | 0.1334±0.2638 | 0.2831±0.3248 | 0.4521±0.6678 |
| *Haemophilus*(%) | 0.6562±2.7976 | 0.1747±0.8758 | 0.2889±0.9146 | 0.2251±0.7442 | 0.2811±1.1920 | 0.1500±0.4822 |
| *Barnesiella*(%) | 0.0526±0.1511 | 0.3616±1.5311 | 0.5161±1.0126 | 0.0157±0.0726 | 0.2917±0.7234 | 0.5841±1.3650 |
| *Phascolarctobacterium*  (%) | 0.6179±2.5672 | 0.1278±0.3538 | 0.2476±0.4101 | 0.2143±0.4049 | 0.1626±0.2796 | 0.2453±0.4720 |
| *Clostridium XlVb*(%) | 0.0950±0.1996 | 0.4861±1.1956 | 0.1095±0.1596 | 0.3840±1.4257 | 0.2020±0.3333 | 0.0935±0.1077 |
| *Erysipelotrichaceae*  *_incertae_sedis*(%) | 0.6624±2.4622 | 0.0786±0.1763 | 0.1068±0.3882 | 0.2731±0.3659 | 0.0834±0.1673 | 0.0409±0.1147 |
| *Parasutterella*(%) | 0.1050±0.3720 | 0.4488±1.4995 | 0.1339±0.5199 | 0.0903±0.1999 | 0.1823±0.6273 | 0.0835±0.2761 |
| *Intestinimonas*(%) | 0.1518±0.2602 | 0.1098±0.1275 | 0.2700±0.2547 | 0.1146±0.3353 | 0.2214±0.4120 | 0.2491±0.2070 |
| *Eubacterium*(%) | 0.0803±0.1821 | 0.2039±0.4221 | 0.1732±0.3752 | 0.1274±0.4694 | 0.2283±0.5582 | 0.2291±0.5938 |
| *Megamonas*(%) | 0.0235±0.1121 | 0.2173±1.0067 | 0.1647±0.6683 | 0.2011±1.0706 | 0.0986±0.5195 | 0.0459±0.1277 |
| *Acetanaerobacterium*  (%) | 0.0726±0.2118 | 0.0565±0.2202 | 0.3776±0.7065 | 0.1214±0.6433 | 0.0466±0.1787 | 0.5350±0.9961 |
| *Eggerthella*(%) | 0.3262±0.4144 | 0.0692±0.0853 | 0.0534±0.1021 | 0.2629±0.4191 | 0.1009±0.1583 | 0.0676±0.1406 |
| *Alloprevotellav*(%) | 0.0082±0.0463 | 00478±0.2210 | 0.3139±0.7685 | 0.0040±0.0189 | 0.0343±0.1925 | 0.0559±0.1820 |
| *Citrobacter*(%) | 0.3044±1.0503 | 0.1006±0.1825 | 0.0350±0.1252 | 0.0891±0.4139 | 0.3649±1.1210 | 0.4706±1.8058 |
| *Oscillibacter*(%) | 0.0450±0.8910 | 0.0767±0.1916 | 0.2695±0.3470 | 0.0523±0.1509 | 0.0871±0.1068 | 0.1756±0.2229 |
| *Turicibacter*(%) | 0.1556±0.4287 | 0.1065±0.3008 | 0.0576±0.1868 | 0.6760±1.4007 | 0.0951±0.2573 | 0.1091±0.3208 |
| *Olsenella*(%) | 0.0132±0.0447 | 0.1343±0.7892 | 0.1355±0.3519 | 0.0386±0.0920 | 0.0366±0.1552 | 0.1456±0.4865 |
| *Senegalimassilia*(%) | 0.0424±0.1343 | 0.0496±0.2228 | 0.1958±0.3403 | 0.0023±0.0135 | 0.0054±0.0233 | 0.5118±1.4314 |
| *Paraprevotella*(%) | 0.0012±0.0033 | 0.1296±0.8825 | 0.1505±0.3945 | 0.0043±0.0114 | 0.0434±0.1868 | 0.0853±0.1690 |
| *Slackia*(%) | 0.0068±0.0224 | 0.0173±0.0627 | 0.2129±0.3514 | 0.0003±0.0017 | 0.0054±0.0260 | 0.2497±0.5192 |
| *Weissella*(%) | 0.0376±0.2003 | 0.1163±0.4394 | 0.0453±0.1523 | 0.0226±0.0703 | 0.0114±0.0276 | 0.2635±0.8340 |
| *Actinomyces*(%) | 0.0612±0.0622 | 0.0735±0.1055 | 0.0789±.0.1045 | 0.1677±0.2160 | 0.1100±0.2894 | 0.0412±0.0440 |
| *Desulfovibrio*(%) | 0.0188±0.0560 | 0.0392±0.1958 | 0.1092±0.3128 | 0.0023±0.0077 | 0.0849±0.3245 | 0.0488±0.2216 |
| *Sutterella*(%) | 0.0009±0.0038 | 0.0847±0.3724 | 0.0816±0.1833 | 0.0040±0.0188 | 0.0342±0.1925 | 0.0559±0.1820 |
| *Sporobacter*(%) | 0.0094±0.0201 | 0.0445±0.0890 | 0.1203±0.1810 | 0.0111±0.0441 | 0.0889±0.2566 | 0.1085±0.1672 |
| *Methanobrevibacter*(%) | 0.0238±0.0859 | 0.0016±0.0055 | 0.1397±0.3884 | 0.0146±0.0457 | 0.0097±0.0319 | 0.1406±0.3615 |
| *Terrisporobacter*(%) | 0.0776±0.2486 | 0.0171±0.0492 | 0.0816±0.2632 | 0.0471±0.14156 | 0.0131±0.0487 | 0.0212±0.0524 |
| *Butyricimonas*(%) | 0.0029±0.0091 | 0.0337±0.0786 | 0.1118±0.1832 | 0.0114±0.0417 | 0.0620±0.1510 | 0.1068±0.2626 |
| *Lactococcus*(%) | 0.0297±0.1290 | 0.0390±0.1548 | 0.0621±0.2136 | 0.0014±0.0036 | 0.0431±0.1335 | 0.2041±1.0707 |
| *Bilophila*(%) | 0.0100±0.0246 | 0.0382±0.0930 | 0.0850±0.1610 | 0.0246±0.0443 | 0.0660±0.1472 | 0.0777±0.1217 |
| *Granulicatella*(%) | 0.0441±0.0457 | 0.0524±0.0805 | 0.0311±0.0595 | 0.1966±0.4778 | 0.0623±0.1374 | 0.0412±0.1112 |
| *Vampirovibrio*(%) | 0.0018±0.0076 | 0 | 0.1108±0.3498 | 0 | 0 | 0.1009±0.5794 |
| *Rothia*(%) | 0.0391±0.0672 | 0.0369±0.1253 | 0.0218±0.0338 | 0.3194±1.0781 | 0.0154±0.0209 | 0.0268±0.0604 |
| *Adlercreutzia*(%) | 0.0465±0.2062 | 0.0198±0.0869 | 0.0295±0.0986 | 0.0069±0.0241 | 0.0177±0.0735 | 0.0006±0.0034 |
| *Intestinibacter*(%) | 0.0085±0.0165 | 0.0088±0.0180 | 0.0561±0.2428 | 0.0449±0.1053 | 0.0137±0.0256 | 0.0076±0.0216 |
| *Gemella*(%) | 0.0400±0.0484 | 0.0259±0.0489 | 0.0195±0.0381 | 0.2557±0.6374 | 0.0277±0.0522 | 0.0232±0.0550 |
| *Saccharibacteria_genera_*  *incertae_sedis*(%) | 0.0141±0.0183 | 0.0427±0.0484 | 0.0203±0.0301 | 0.0677±0.1102 | 0.0589±0.0985 | 0.0224±0.0361 |
| *Peptoniphilus*(%) | 0.0062±0.0227 | 0.0304±0.1670 | 0.0384±0.1107 | 0.0257±0.1314 | 0.0163±0.0495 | 0.0121±0.0242 |
| *Gordonibacter*(%) | 0.0432±0.1193 | 0.0155±0.0364 | 0.0174±0.0236 | 0.0077±0.0133 | 0.0166±0.0272 | 0.0312±0.0340 |
| *Peptococcus*(%) | 0 | 0.0010±0.0047 | 0.0695±0.2718 | 0.0354±0.2044 | 0.0043±0.0174 | 0.0018±0.0046 |
| *Succinivibrio*(%) | 0.0009±0.0051 | 0.0071±0.0500 | 0.0616±0.2854 | 0 | 0.0034±0.0203 | 0.0332±0.1938 |
| *Pseudoflavonifractor*  (%) | 0.0065±0.0186 | 0.0135±0.0269 | 0.0503±0.0500 | 0.0086±0.0283 | 0.0329±0.0777 | 0.0509±0.0546 |
| *Porphyromonas*(%) | 0.0003±0.0017 | 0.0173±0.0932 | 0.0526±0.1505 | 0.0029±0.0138 | 0.0054±0.0174 | 0.0218±0.0504 |
| *Clostridium III*(%) | 0.0015±0.0086 | 0.0569±0.3529 | 0.0045±0.0159 | 0.0034±0.0171 | 0.0014±0.0069 | 0.0300±0.0639 |
| *Megasphaera*(%) | 0.0071±0.0247 | 0.0112±0.0315 | 0.0826±0.4358 | 0.0014±0.0036 | 0.0431±0.1335 | 0.2041±1.0707 |
| *Anaerotruncus*(%) | 0.0074±0.0297 | 0.0088±0.0188 | 0.0247±0.0354 | 0.0103±0.025 | 0.0397±0.0798 | 0.0129±0.0233 |
| *Parvibacter*(%) | 0 | 0.0008±0.0034 | 0.0518±0.2899 | 0 | 0.0017±0.0057 | 0.0038±0.0144 |
| *Mogibacterium*(%) | 0.0112±0.0264 | 0.0031±0.0121 | 0.0403±0.1040 | 0.0186±0.0399 | 0.0080±0.0227 | 0.1221±0.5488 |
| *Atopobium*(%) | 0.0121±0.0264 | 0.0194±0.0340 | 0.0276±0.0441 | 0.0160±0.0326 | 0.0271±0.0555 | 0.0097±0.0131 |
| *Anaeroglobus*(%) | 0.0094±0.0515 | 0.0300±0.0271 | 0.0182±0.1120 | 0 | 0.0006±0.0024 | 0.0036±0.0206 |
| *Enterorhabdus*(%) | 0.0029±0.01382 | 0 | 0.0476±0.1100 | 0.0009±0.0051 | 0 | 0.0409±0.0999 |
| *Howardella*(%) | 0.0041±0.0208 | 0.0198±0.0932 | 0.0255±0.0551 | 0.0066±0.0306 | 0.0003±0.0017 | 0.0159±0.0371 |
| *Eisenbergiella*(%) | 0.0129±0.0343 | 0.0208±0.0592 | 0.0232±0.0711 | 0.0214±0.0585 | 0.0220±0.0846 | 0.0438±0.1172 |
| *Faecalitalea*(%) | 0.0524±0.3053 | 0 | 0 | 0.0003±0.0017 | 0.0049±0.0287 | 0 |
| *Holdemania*(%) | 0.0109±0.0210 | 0.0192±0.0611 | 0.0224±0.0355 | 0.0117±0.0316 | 0.0200±0.0357 | 0.0209±0.0427 |
| *Odoribacter*(%) | 0.0018±0.0046 | 0.0092±0.0218 | 0.0400±0.0502 | 0.0003±0.0017 | 0.0317±0.0916 | 0.0553±0.1256 |
| *Leuconostoc*(%) | 0.0024±0.0061 | 0.0131±0.0200 | 0.0258±0.0523 | 0.0791±0.2552 | 0.0134±0.0291 | 0.0400±0.0592 |
| *Anaerovorax*(%) | 0.0074±0.0297 | 0.0088±0.0188 | 0.0247±0.0221 | 0.0083±0.0167 | 0.0217±0.0488 | 0.0374±0.0701 |
| *Allisonella*(%) | 0.0068±0.0233 | 0.0094±0.0313 | 0.0221±0.0715 | 0.0011±0.0068 | 0.0043±0.0112 | 0.0432±0.1349 |
| *Coprobacillus*(%) | 0.0053±0.0105 | 0.0110±0.0417 | 0.0213±0.0505 | 0.0651±0.3458 | 0.0046±0.0115 | 0.0282±0.1318 |
| *Solobacterium*(%) | 0.0065±0.0098 | 0.0198±0.0386 | 0.0055±0.0111 | 0.0291±0.0673 | 0.0669±0.2643 | 0.0065±0.0104 |
| *Ezakiella*(%) | 0.0003±0.0017 | 0.0100±0.0571 | 0.0216±0.0738 | 0.0063±0.0321 | 0.0054±0.0287 | 0.0032±0.0101 |
| *Butyrivibrio*(%) | 0.0024±0.0078 | 0.0012±0.0044 | 0.0232±0.0556 | 0.0029±0.0079 | 0.0260±0.1366 | 0.0171±0.0448 |
| *Gardnerella*(%) | 0.0200±0.1149 | 0 | 0.0079±0.0278 | 0.0014±0.0085 | 0.0006±0.0024 | 0.0053±0.0244 |
| *Mitsuokella*(%) | 0 | 0.0224±0.1203 | 0.0050±0.0230 | 0 | 0.0111±0.0575 | 0.0532±0.2544 |
| *Coriobacterium*(%) | 0.0032±0.0088 | 0.0024±0.0063 | 0.0158±0.0339 | 0.0017±0.0101 | 0.0060±0.0148 | 0.0094±0.0152 |
| *Christensenella*(%) | 0.0056±0.0111 | 0.0051±0.0124 | 0.0134±0.0263 | 0.0023±0.0106 | 0.0063±0.0121 | 0.0203±0.0425 |
| *Cronobacter*(%) | 0.0059±0.0235 | 0.0147±0.0418 | 0.0005±0.0023 | 0.0029±0.0153 | 0.0063±0.0146 | 0.0215±0.1131 |
| *Peptostreptococcus*(%) | 0.0041±0.0099 | 0.0039±0.0076 | 0.0139±0.0523 | 0.0157±0.0333 | 0.0020±0.0053 | 0.0053±0.0133 |
| *Anaerococcus*(%) | 0.0021±0.0104 | 0.0037±0.0174 | 0.0145±0.0378 | 0.0097±0.0438 | 0.0043±0.0124 | 0.0079±0.0313 |
| *Saccharofermentans*(%) | 0.0021±0.0120 | 0.0031±0.0172 | 0.0150±0.0512 | 0.0037±0.0186 | 0.0031±0.0170 | 0.0024±0.0085 |
| *Oxalobacter*(%) | 0.00120.0041 | 0.0078±0.0347 | 0.0116±0.0230 | 0.0003±0.0017 | 0.0074±0.0196 | 0.0288±0.08686 |
| *Allobaculum*(%) | 0 | 0.0149±0.1043 | 0.0005±0.0023 | 0 | 0 | 0.0018±0.0103 |
| *Coprobacter*(%) | 0 | 0.0004±0.0020 | 0.0197±0.0634 | 0 | 0.0069±0.0215 | 0.0962±0.5176 |
| *Alloscardovia*(%) | 0.0018±0.0087 | 0.0018±0.0076 | 0.0129±0.0746 | 0±0 | 0.0006±0.0024 | 0.0006±0.0024 |
| Parasporobacterium(%) | 0.0038±0.0191 | 0.0022±0.0143 | 0.0071±0.0438 | 0.0029±0.0086 | 0.0094±0.04759 | 0 |
| *Hydrogenoanaerobacterium*  (%) | 0.0015±0.0044 | 0.0045±0.0176 | 0.0071±0.0438 | 0.0023±0.0065 | 0.0026±0.0078 | 0.0094±0.0116 |
| *Corynebacterium*(%) | 0.0059±0.0118 | 0.0031±0.0080 | 0.0029±0.0061 | 0.0229±0.0444 | 0.0026±0.0061 | 0.0024±0.0043 |
| *Parvimonas*(%) | 0.0029±0.0063 | 0.0018±0.0049 | 0.0068±0.1630 | 0.0089±0.0171 | 0.0066±0.0181 | 0.0044±0.0140 |
| *Vagococcus*(%) | 0.0003±0.0017 | 0.0006±0.0024 | 0.0124±0.762 | 0.0003±0.0017 | 0±0 | 0.0024±0.0092 |
| *Enterobacter*(%) | 0.0009±0.0038 | 0.0096±0.0350 | 0.0016±0.0037 | 0.0277±0.1554 | 0.0040±0.0109 | 0.0041±0.0118 |
| *Scardovia*(%) | 0.0006±0.0024 | 0.0010±0.0031 | 0.0089±0.0374 | 0.0046±0.0131 | 0.0006±0.0024 | 0.0006±0.0024 |
| *Oribacterium*(%) | 0.0044±0.0121 | 0.0043±0.0094 | 0.0013±0.0053 | 0.0129±0.0364 | 0.0080±0.0173 | 0.0012±0.0033 |
| *Mycoplasma*(%) | 0.0003±0.0017 | 0.0133±0.0453 | 0.0003±0.0016 | 0±0 | 0.0017±0.0101 | 0.0018±0.0087 |
| *Anaerobacter*(%) | 0.0003±0.0024 | 0.0002±0.0014 | 0.0061±0.0308 | 0.0029±0.0086 | 0.0006±0.0024 | 0.0038±0.0144 |
| *Catabacter*(%) | 0.0015±0.0044 | 0.0018±0.0044 | 0.0055±0.0143 | 0.0017±0.0051 | 0.0029±0.0067 | 0.0044±0.0113 |
| *Finegoldia*(%) | 0.0015±0.0044 | 0.0022±0.0105 | 0.0039±0.0179 | 0.0163±0.0726 | 0.0003±0.0017 | 0.0026±0.0105 |
| *Abiotrophia*(%) | 0.0026±0.0105 | 0.0027±0.0057 | 0.0026±0.0101 | 0.1734±0.5714 | 0.0086±0.0228 | 0.0024±0.0089 |
| *Methanoculleus*(%) | 0 | 0.0067±0.0175 | 0 | 0.0034±0.0128 | 0.0643±0.3319 | 0.0006±0.0034 |
| *Aestuariispira*(%) | 0.0003±0.0017 | 0.0031±0.0200 | 0.0042±0.0141 | 0 | 0 | 0.0009±0.0038 |
| *Morganella*(%) | 0 | 0.0014±0.0087 | 0.0037±0.0158 | 0 | 0.1780±1.0237 | 0 |
| *Tepidimicrobium*(%) | 0 | 0.0018±0.0129 | 0.0039±0.0197 | 0 | 0.0006±0.0024 | 0.0003±0.0017 |
| *Asteroleplasma*(%) | 0 | 0.0035±0.0243 | 0.0018±0.0114 | 0 | 0 | 0.0003±0.0017 |
| *Anaerofilum*(%) | 0.0021±0.0088 | 0.0008±0.0045 | 0.0021±0.0047 | 0.0026±0.0107 | 0.0055±0.0144 | 0.0053±0.0146 |
| *Negativicoccus*(%) | 0 | 0.0004±0.0029 | 0.0039±0.0173 | 0.0023±0.0135 | 0.0009±0.0051 | 0.0003±0.0017 |
| *Staphylococcus*(%) | 0.0003±0.0017 | 0.0014±0.0061 | 0.0021±0.0062 | 0.0729±0.2755 | 0 | 0.0065±0.0294 |
| *Hungatella*(%) | 0.0003±0.0017 | 0.0016±0.0090 | 0.0021±0.0114 | 0.0003±0.0017 | 0 | 0.0003±0.0017 |
| *Defluviitalea*(%) | 0.0009±0.0038 | 0.0006±0.0032 | 0.0029±0.0061 | 0.0003±0.0017 | 0.0020±0.0076 | 0.0018±0.0046 |
| *Sneathia*(%) | 0.0003±0.0017 | 0 | 0.0037±0.0105 | 0.0009±0.0051 | 0 | 0.0009±0.0051 |
| *Delftia*(%) | 0.0018±0.0103 | 0.0018±0.0115 | 0.0008±0.0036 | 0.0120±0.0420 | 0.0003±0.0017 | 0.0003±0.0017 |
| *Lachnoanaerobaculum*  (%) | 0.0009±0.0038 | 0.0020±0.0074 | 0.0003±0.0016 | 0.0166±0.0524 | 0.0026±0.0061 | 0.0015±0.0070 |
| *Anaerofustis*(%) | 0.0035±0.0098 | 0.0002±0.0014 | 0 | 0.0017±0.0051 | 0.0003±0.0017 | 0 |
| *Murdochiella*(%) | 0.0003±0.0017 | 0.0004±0.0029 | 0.0024±0.0071 | 0.0017±0.0101 | 0.0006±0.0024 | 0.0006±0.0024 |
| *Cloacibacillus*(%) | 0.0018±0.0076 | 0.0016±0.0075 | 0 | 0.0026±0.0104 | 0.0011±0.0040 | 0.0003±0.0017 |
| *Raoultella*(%) | 0.0012±0.0054 | 0.0012±0.0053 | 0.0003±0.0016 | 0 | 0.0126±0.0624 | 0.0015±0.0036 |
| *Anoxybacillus*(%) | 0.0003±0.0017 | 0.0006±0.0032 | 0.0016±0.0097 | 0.0011±0.0040 | 0.0009±0.0028 | 0.0077±0.0429 |
| *Thauera*(%) | 0.0038±0.0065 | 0 | 0 | 0.0057±0.0288 | 0 | 0 |
| *Acidaminococcus*(%) | 0.0003±0.0017 | 0.0035±0.0229 | 0 | 0 | 0.0003±0.0017 | 0 |
| *Pyramidobacter*(%) | 0.0003±0.0017 | 0.0012±0.0048 | 0.0016±0.0059 | 0.0006±0.0024 | 0.0034±0.0159 | 0 |
| *Methanomassiliicoccus*(%) | 0.0003±0.0017 | 0.0016±0.0043 | 0 | 0.0037±0.0100 | 0.0171±0.0928 | 0.0006±0.0034 |
| *Bulleidia*(%) | 0 | 0.0014±0.0046 | 0.0008±0.0036 | 0.0009±0.0028 | 0.0005±0.0034 | 0.0018±0.0063 |
| *Caulobacter*(%) | 0.0015±0.0086 | 0.0004±0.0020 | 0 | 0.0291±0.0817 | 0.0054±0.0321 | 0 |
| *Pseudomonas*(%) | 0.0012±0.0041 | 0.0006±0.0024 | 0 | 0.7526±2.7471 | 0.0157±0.0401 | 0.00244±0.0055 |
| *Subdoligranulum*(%) | 0.0009±0.0051 | 0.0004±0.0020 | 0 | 0 | 0.0003±0.0017 | 0.0003±0.0017 |
| *Hafnia*(%) | 0.0003±0.0017 | 0.0012±0.0073 | 0.0003±0.0016 | 0.0143±0.0846 | 0.0017±0.0062 | 0.0029±0.0155 |
| *Leclercia*(%) | 0 | 0.0002±0.0014 | 0.0016±0.0097 | 0.00029±0.0017 | 0.0006±0.0024 | 0 |
| *Burkholderia*(%) | 0.0015±0.0044 | 0.0014±0.0046 | 0.0005±0.0023 | 0.00029±0.0017 | 0.0094±0.0147 | 0.0006±0.0024 |
| *Asaccharobacter*(%) | 0.0009±0.0051 | 0.0004±0.0002 | 0 | 0.00029±0.0017 | 0 | 0.0003±0.0017 |
| *Murimonas*(%) | 0.0003±0.0017 | 0.0008±0.0034 | 0.0005±0.0032 | 0 | 0.0006±0.0024 | 0.0018±0.0063 |
| *Sphingomonas*(%) | 0.0006±0.0034 | 0.0002±0.0014 | 0.0011±0.0031 | 0.0120±0.0282 | 0.0037±0.0069 | 0.0012±0.0033 |
| *Campylobacter*(%) | 0 | 0 | 0.0021±0.0062 | 0.0026±0.0136 | 0.0003±0.0017 | 0.0024±0.0121 |
| *Roseibacillus*(%) | 0.0012±0.0054 | 0 | 0.0008±0.0027 | 0.0114±0.0406 | 0±0 | 0.0021±0.0054 |
| *Mobiluncus*(%) | 0 | 0.0002±0.0014 | 0.0011±0.0051 | 0 | 0.0006±0.0024 | 0.0009±0.0038 |

Values are expressed as mean±SD. Abbreviations: LHH, the Han HPs from low-altitude (Wuhan: 13 m); MHH, the Han HPs living at middle-altitude (Xining: 2260 m); HTH, the Tibetan HPs residing at high-altitude (Qinghai-Tibet Plateau: 3600–4500 m); LHN, the Han HIs from low-altitude (Wuhan: 13 m); MHN, the Han HIs living at middle-altitude (Xining: 2260 m); HTN, the Tibetan HIs residing at high-altitude (Qinghai-Tibet Plateau: 3600–4500 m).
